# Self-Medication Practices During the COVID-19 Pandemic Among the Adult Population in the Eastern Region of the Kingdom of Saudi Arabia

**DOI:** 10.7759/cureus.40505

**Published:** 2023-06-16

**Authors:** Abdullah Almaqhawi, Mahdi Alhamad, Baqer Albaqshi, Mohammed Alquraini, Musawi Altaha, Hassan Alhussain, Raed Alfayez, Sayed Ibrahim Ali

**Affiliations:** 1 Family and Community Medicine, College of Medicine, King Faisal University, Hofuf, SAU

**Keywords:** public health, kingdom of saudi arabia, adult population, covid-19 pandemic, self-medication

## Abstract

Background: Self-medication is vital to public health because it has an impact on people's health and the current healthcare system, both positively and negatively. During public health catastrophes like the COVID-19 disease, this is particularly true.

Aim: This study aimed to examine the behavioral response of the community with regard to self-medication during the COVID-19 pandemic in the eastern region of the Kingdom of Saudi Arabia.

Methods: During the COVID-19 outbreak from March to September 2020, a cross-sectional online survey of 398 participants using structured questionnaires was conducted to observe knowledge, prevalence, patterns, and sources of self-medication among the respondents in the eastern region of the Kingdom of Saudi Arabia.

Results: The percentage of respondents who had heard about self-medication was 50.5%, and those who practiced self-medication during COVID-19 were 43.7% of the respondents. Regarding knowledge, 60.3% had a low overall knowledge level versus 39.7% who had a high knowledge level. Most of those who practiced self-medication took medication based on their own decision (34.4%). The most frequently used drugs during the outbreak were analgesics (43.5%) and vitamins (24.9%). Only 1% of participants reported using anti-malaria drugs (hydroxychloroquine). The most common reasons for self-medication practices were having a mild illness (30.4%), followed by fear of infection (26.6%). The symptoms for which the respondents took self-medication were headache (29.6%), cough (26.6%), and fever (24.6%).

Conclusion: Our investigation showed a low level of knowledge about self-medication and a considerable level of self-medication practices. Therefore, self-medication may be minimized with ongoing awareness-raising and sensitization.

## Introduction

Self-medication (SM) is defined by the World Health Organization (WHO) as "the selection and use of medicines by individuals (or a member of the individual’s family) to treat self-recognized or self-diagnosed conditions or symptoms [1]. Globally, self-medication is a major concern, affecting both developed and developing countries [2,3]. Several studies have shown that SM is common, with a worldwide prevalence of 32.5-81.5% [4]. Analgesics, antipyretics, antitussives, antidiarrheals, calcium and vitamin supplements, anabolic steroids, sedatives, some antibiotics, and many herbal and homeopathic remedies are the most often self-prescribed medications [2]. The COVID-19 lockdown led to self-reliance, with fake news exacerbating self-medication concerns [5,6]. Additionally, the extensive news exposures associated with any study (in vitro, pre-clinical, or clinical) that shed light on preventive or treating options exacerbated the issue as well [7].

Common reasons for self-medication included delays in accessing health care, socio-cultural beliefs, relatively high hospital costs, expertise in treating similar symptoms, easy medical availability, poor regulatory practice, the urgency of feeling relieved, and advice from friends and the media [1]. The WHO emphasized that reasonable practice of self-medication can help to prevent and treat certain minor diseases at an affordable cost, but otherwise, it will be a waste of resources and may cause pathogen resistance and serious health risks with adverse drug reactions and prolonged morbidity [8,9]. The psychological distress caused by the COVID-19 pandemic was positively associated with self-medication [10].

A recent Google Trends study has shown an upward trend regarding self-medication during the COVID-19 pandemic. This study has shown an increasing number of searches worldwide for self-medication and would be an indication of increasing self-medication interests around the globe since the declaration of the pandemic [11]. Several studies have been conducted recently to assess SM practices during the COVID-19 pandemic. A survey of members of the Australian population found that around one in five people self-medicate with antibiotics to protect themselves against COVID-19 [10]. In Dhaka, Bangladesh, the prevalence of SM was 88.33% during the COVID-19 outbreak [12]. Also, SM was found to be a major health issue in Peru, particularly during the COVID-19 pandemic. Different drugs have been used without adequate scientific evidence for COVID-19-related respiratory symptoms [13]. Another study conducted in Poland showed that nearly half of the respondents had at least one behavior during the lockdown associated with improper self-medication, and some of them had never committed such behaviors before the lockdown [14].

In Saudi Arabia, the number of COVID-19 cases started to rise significantly around May 16, 2020, at 2840, or 81.58 per million, while the number of currently infected cases peaked at 28728, or 825.19 per million, on May 25, 2020 [15]. Self-medication was found to be common in Saudi Arabia. Sources of information, knowledge, and perception were inappropriate and inadequate. Antibiotics and analgesics were the most common prescription medications dispensed without a prescription. Nearly half of the drugs that consumers purchased without a prescription from community pharmacies were prescription-only medicines [16]. Moreover, in Saudi Arabia, a study revealed that 77.6% of pharmacies dispensing antibiotics do so without a prescription, and almost 95% do so without the patient's request [17]. Additional studies on self-medication patterns and their impact in a pandemic situation are mandatory, and effective information on the use and hazards of medicines in the context of a pandemic is crucial [18].

Therefore, our study aims to examine the behavioral response of the community with regard to self-medication during the COVID-19 pandemic in the eastern region of the Kingdom of Saudi Arabia.

## Materials and methods

In this online cross-sectional survey, a descriptive, non-experimental research design was used to examine self-medication practices from March to September 2020, the period during the nationwide lockdown and a surge in the number of positive COVID-19 cases. The inclusion criteria were adult Saudi citizens of the eastern region of the Kingdom of Saudi Arabia who were at least 18 years of age and residents of the region. The sample size was 385 using the formula n=Z2 pq/E2, where the margin of error (E) equals 0.05. The confidence level (Za/2) was 95%, which equals 1.96. The expected proportion (p) of adults equals 0.5; the actual sample size was 398 randomly selected participants. In order to learn more about how the general public felt about taking over-the-counter medicines during the COVID-19 outbreak, respondents with medical knowledge or background (e.g., medical school graduates, medical practitioners, nurses, or medical researchers) were excluded from the survey. The response frequencies were noted in a datasheet and tracked in relation to demographic data, information sources, clinical symptoms, and the status of the COVID-19 test results. Face and content validity techniques were used to create and validate the questionnaire. Face validity was achieved by administering the draft questionnaire to a few citizens who met the inclusion criteria in the Eastern region in order to determine whether the response appeared meaningful, well-designed, and/or a good measure of the construct to an innocent participant. The questionnaire was further improved and altered using the data gathered from this exercise. Three independent researchers from the fields of social statistics, community medicine, and public health evaluated the questionnaire's appropriateness, clarity, coverage, and relevance to the study as part of the content validity process. The incorporated draft questionnaire was rewritten to remove vagueness and eliminate questions that were asked more than once. The reliability of the questionnaire was calculated by using Cronbach’s alpha test, and the result showed a Cronbach’s alpha value of 0.837, indicating that the questionnaire was highly reliable.

Statistical analysis

Descriptive statistics were used to summarize the responses. Frequency distributions were generated to show the proportion of participants who self-medicated during the lockdown period, the types of medications used, and the sources of information they relied on. Means and standard deviations (SD) were calculated to describe the age; frequencies and percentages were used to describe the categorical variables. A Chi-square test was performed to explore the relationship between self-medication practices and the demographic characteristics of the participants, such as age, gender, and education level.

All statistical analyses were conducted using Statistical Package for the Social Sciences (SPSS) version 26.0 (IBM Corp., Armonk, NY, USA), and a p-value of less than 0.05 was considered statistically significant.

## Results

A total of 398 adult participants completed the study questionnaire. Participants' ages ranged from 18 to more than 65 years old, with a mean age of 39.5 ± 13.7 SD years old. A total of 245 (61.6%) participants were female. Regarding marital status, 252 (63.3%) were married. Considering education level, 202 (50.8%) had bachelor's degrees, and 33 (8.3%) had post-graduate degrees. Considering monthly income, it was less than 4000 Saudi Riyals (SAR) among 213 (53.5%) participants and more than 12000 SAR among 15.6%. Table 1 represents the socio-demographic variables for the studied participants.

**Table 1:** The sociodemographic variables for the studied participants SAR: Saudi Riyal

		n	%
Age (in years)	18-29	166	41.7
	30-49	191	48.0
	50-65	38	9.5
	above 65	3	0.8
Gender	Male	153	38.4
	Female	245	61.6
Marital status	Single	146	36.7
	Married	252	63.3
Educational level	Elementary school	5	1.3
	Intermediate school	14	3.5
	Secondary education	102	25.6
	Diploma	42	10.6
	Bachelor's degree	202	50.8
	Higher diploma	7	1.8
	Master's degree	22	5.5
	Ph.D.	4	1.0
Income (SAR per month)	Less than 4000 SAR	213	53.5
	4000-7999 SAR	66	16.6
	8000-11999 SAR	57	14.3
	more than 12000 SAR	62	15.6

Table 2 shows the knowledge of the adult population in the eastern region regarding self-medication. Exactly 201 (50.5%) heard about self-medication, 174 (43.7%) practiced self-medication, 155 (38.9%) thought that self-medication practices result in harmful effects, and 280 (70.4%) refused that self-medication is better than seeking medical consultation. The knowledge categories were calculated based on the knowledge score mean (3.2), with low knowledge below 3.2 and high knowledge above 3.2. Totally, 240 (60.3%) had a low overall knowledge level, versus 158 (39.7%) who had a high knowledge level.

**Table 2:** The frequencies and percentages of knowledge on self-medication

		n	%
Knowledge categories	Low knowledge	240	60.3
	High knowledge	158	39.7
Have you ever heard about self-medication?	Yes	201	50.5
	No	166	41.7
	I don't know	31	7.8
Have you ever practiced self-medication?	Yes	174	43.7
	No	224	56.3
Can self-medication practices result in harmful effects?	Yes	155	38.9
	No	116	29.1
	I don't know	127	31.9
Is self-medication better than seeking medical consultation?	Yes	38	9.5
	No	280	70.4
	I don't know	80	20.1

Table 3 represents the reasons and symptoms for practicing self-medication by adults in the eastern region during the COVID-19 pandemic. As for the reason(s) for self-medication, the most reported were having a mild illness (30.4%), followed by fear of infection (26.6%) and protection against COVID-19 (18.6%), while 29.4% did not self-medicate. Considering the symptom(s) for which you use the medication, the most frequent included headache (29.6%), cough (26.6%), fever (24.6%), sore throat (21.6%), and runny nose (20.6%).

**Table 3:** Reasons for practicing self-medication

		N	%
What was your reason(s) for practicing self-medication?	Cost saving	35	8.8
	Lack of trust in the doctor	15	3.8
	Mild illness	121	30.4
	Fear of infection or contact while visiting healthcare facilities	106	26.6
	Delay in receiving treatment at health facilities	38	9.5
	Proximity of the pharmacy to home	31	7.8
	COVID-19 infection (positive result)	38	9.5
	I didn't self-medicate	117	29.4
	Protection against COVID-19	74	18.6
Indicate the symptom(s) for which you used the medication	Cough	106	26.6
	Runny nose	82	20.6
	Nasal congestion	64	16.1
	Breathing difficulty	20	5
	Sore throat	86	21.6
	Fever	98	24.6
	Muscle pain	61	15.3
	Headache	118	29.6
	Vomiting	7	1.8
	Diarrhea	27	6.8
	Skin problem	35	8.8
	I used the drug even though I did not have any symptoms	13	3.3
	I didn't use the drugs	112	28.1

Table 4 shows the pattern of self-medication among adults in the eastern region during the COVID-19 pandemic. As for self-treating frequency during the COVID-19 pandemic, 32.9% reported one to three times, 7% reported more than 10 times, and 15.6% never did. The most commonly used medications were analgesics (43.5%), followed by vitamins (24.9%), and antibiotics (11.1%). Selection of medication based on their own decision among 24.4% of participants, opinions of friends or family members (21.9%), and social media and the internet (14.6%). The most reported sources of medications were pharmacies (22.9%), being available at home from previous prescriptions (19.8%), and being available at home from family (8%).

**Table 4:** The pattern of self-medication among adults in the eastern region during the COVID-19 pandemic NSAIDs: non-steroidal anti-inflammatory drugs

		N	%
How many times did you treat yourself by self-medication during the COVID-19 pandemic?	.00	62	15.6
	1-3 times	131	32.9
	4-5 times	33	8.3
	6-10 times	16	4
	More than 10 times	28	7
	I did not self-medicate	128	32.2
What did you use for self-medication?	Antibiotics	44	11.1
	Antihistamine	40	10.1
	Analgesics	173	43.5
	NSAIDs	25	6.3
	Corticosteroids	5	1.3
	Anti-malaria medicines	4	1
	Vitamins	99	24.9
	Herbal products	12	3
	I did not use any medicines	106	26.6
Your selection of medication is based on	My own experience	137	34.4
	Opinions of friends or family members	87	21.9
	Social media and the internet	58	14.6
	Previous prescription by a doctor	57	14.3
	Recommendation by the pharmacist	31	7.8
	I did not use any medicines	118	29.6
Your source of medications	Pharmacy (over-the-counter medicines)	91	22.9
	Available at home from your previous prescription	79	19.8
	Available at home from a family member's previous prescription	32	8
	Workers in health facilities	16	4
	I did not use any medication	116	29.1
	Herbal shop	2	0.5

Considering relations, a total of 73.7% of participants aged 50-65 years had a low knowledge level versus 53% of others aged 18-29 years, with recorded statistical significance (p=.005). Low knowledge level was insignificantly higher among female adults (61.2% vs. 58.8%), married adults (60.7% vs. 59.6%), Saudi adults (60.8% vs. 37.5%), elementary school adults (80% vs. 75% for PhD), and high-income adults (67.7% vs. 55.9% for others with lowest income), with all having an insignificant association (p>0.05 for all).

Age also showed a significant association with "proximity of the pharmacy to home," with the highest frequency at old age above 65 years (100%) (p=.022) and with "I used the drug even though I did not have any symptoms," with the highest frequency in the same age group (100%) (p=.041). Gender was significantly associated with "skin problems,", with the highest frequency among male adults (96.7%) (p=.002). In comparison, marital status showed a significant association with reasons for using self-medication, with the highest rate among the married group (93.7%) (p=.024). Also, marital status showed a significant association with "mild illness", with the highest rate among married adults (74.2%) (p=.009). Educational level was significantly associated with "fear of infection or contact while visiting healthcare facilities," with the highest frequency in the intermediate school group (92.9%) (p=.002). Also, educational level showed a significant association with "I used drugs even though I did not have any symptoms," with the highest frequency among intermediate schools (100%) (p=.002). Income also showed a significant association with "lack of trust in the doctor," with the highest reported frequency among middle-level income participants (4000-7999 SAR; 100%) (p=.002). Gender showed a significant relationship with "analgesics," with the highest percentage of male participants (63.4%) (p=.029), and also with "NSAIDs," with the highest frequency among males (97.4%) (p=.017). Additionally, male adults showed a significantly higher rate of "previous prescription by a doctor" (90.8%) (p=.020) and a source of medication where 28.7% of males reported pharmacy versus 26.2% of females (p=.015). Sixty percent of elementary school participants never treated themselves versus any of the high diploma participants and 50% of others with Ph.D. degrees (p=.001).

Also, 19.6% of participants with low knowledge never self-treated compared to 9.5% of others with high knowledge (p=.002). Also, knowledge level was significantly associated with "analgesics", with the highest frequency among low-knowledge participants (63.8%) (p=.001). Exactly 79.1% of participants with high knowledge reported "I did not practice self-medication" versus 69.6% of those with low knowledge levels (p=.035). Additionally, 72.9% of participants with low knowledge self-selected medication in comparison to 54.4% of others with high knowledge (p=.001). Exactly 89.2% of participants with low knowledge reported "previous prescription by a doctor" versus 80.4% of others (p=.014) and 95% reported "recommendation by the pharmacist" versus 88%, respectively (p=.011).

As for the relationship between knowledge level and demographics, higher knowledge was significantly associated with old age, where 100% had a high knowledge level (p=.005).

## Discussion

This study has revealed some interesting results regarding knowledge, prevalence, patterns, and sources of self-medication during the COVID-19 pandemic among the respondents. One hundred and seventy-four respondents (43.7%) had practiced self-medication with various medicines; 45.2% used self-medication due to fear or protection from infection. Overall, participants exhibited a good attitude toward self-medication but poor knowledge.

In the current study, 245 (61.6%) participants were female, which is consistent with some studies from other regions [19, 20], which show that female participants are more likely to self-medicate than their male peers. On the other hand, there are some studies that suggest male predominance in the same context [21,22]. Also, almost half of the study participants (50.8%) had a bachelor’s degree, which shows similar results to a regional study conducted locally in Saudi Arabia, where over two-thirds of respondents had a university education [23].

A local study had shown that a poor level of knowledge was observed among participants when it came to purchasing prescription-only medications without a prescription and knowing the drug instructions [16]. Similarly, the current study showed low knowledge levels in 60.3% of the participants related to knowledge of the harmful effects of self-medication and the importance of seeking medical consultation instead of self-medication.

Regarding the causes of self-medication, our study has stated that mild illness was the most common reason for self-medication (30.4%), followed by fear of infection (26.6%) and protection against COVID-19 (18.6%), when almost 29.4% practiced self-medication. In the same context, two studies conducted in Peru and Central Saudi Arabia revealed similar results, as mild symptoms like a cold or flu were commonly the main reason for self-medication [13,16], while another study from Bahrain, which is a contiguous country, suggested cost savings as an additional reason favored by 14.9% of the respondents [24]. Also, respondents tend to self-medicate while having certain symptoms, of which fever (37.6%) is the most common in a study done in Bangladesh during the COVID-19 outbreak [20]. Two other studies have stated headache as the most common symptom but with a similar percentage of fever compared to the previously mentioned research [19,24]. Similarly, our study suggests headache (29.6%) as the most common symptom, followed by cough and fever (26.6%) and (24.6%), respectively.

According to a systematic review conducted in Iran about predictors of self-medication [25], friends, relatives, and family, previous prescriptions by physicians, and a pharmacist's recommendation represent the most common sources of self-medication. Different results were shown in the current study, as 22.9% of the respondents reported the pharmacy as the main source, followed by being available at home from previous prescriptions and being available at home from family.

Several studies have discussed the drugs that are utilized in self-medication. One study conducted in Togo during the COVID-19 outbreak described vitamin C (27.6%) as the most commonly used [26]. Two other regional studies that were conducted before the COVID-19 pandemic in 2011 and 2004, respectively, described analgesics as the most commonly used, followed by decongestants, cough preparations, and antibiotics [16,2]. In the current study, analgesics (43.5%) were the most commonly used, followed by vitamins (24.9%) and antibiotics (11.1%).

## Conclusions

The present study provides important insights into self-medication practices among the adult population of the eastern region of the Kingdom of Saudi Arabia during the COVID-19 pandemic. The study found that self-medication practices were prevalent among the respondents, with more than 40% of them reporting self-medication use. The reasons for self-medication included mild illnesses, fear of infection, and protection against COVID-19. However, the study also showed that a significant proportion of respondents had low overall knowledge of self-medication practices, which indicates the need for targeted educational programs to improve public awareness of the risks and benefits of self-medication. Moreover, analgesics and vitamins were the most common medications used, with the majority of respondents obtaining them from pharmacies. The study recommends that healthcare providers emphasize the importance of consulting with healthcare professionals before starting any medication to prevent harm to themselves. Lastly, further research in this area should explore the reasons behind the widespread use of self-medication, assess its consequences, and investigate ways to improve public health and prevent the risks associated with self-medication practices.
